# Left Ventricular Remodeling in Non-syndromic Mitral Valve Prolapse: Volume Overload or Concomitant Cardiomyopathy?

**DOI:** 10.3389/fcvm.2022.862044

**Published:** 2022-04-12

**Authors:** Lobke L. Pype, Philippe B. Bertrand, Bernard P. Paelinck, Hein Heidbuchel, Emeline M. Van Craenenbroeck, Caroline M. Van De Heyning

**Affiliations:** ^1^Department of Cardiology, Antwerp University Hospital, Antwerp, Belgium; ^2^Genetics, Pharmacology and Physiopathology of Heart, Vasculature and Skeleton (GENCOR) Research Group, University of Antwerp, Antwerp, Belgium; ^3^Department of Cardiology, Ziekenhuis Oost-Limburg, Genk, Belgium; ^4^Cardio and Organ Systems (COST) Resarch Group, Hasselt University, Hasselt, Belgium; ^5^Department of Cardiac Surgery, Antwerp University Hospital, Antwerp, Belgium

**Keywords:** mitral valve prolapse, cardiomyopathy, mitral regurgitation, cardiac imaging, echocardiography, cardiac magnetic resonance (CMR) imaging, left ventricular remodeling

## Abstract

Mitral valve prolapse (MVP) is a common valvular disorder that can be associated with mitral regurgitation (MR), heart failure, ventricular arrhythmias and sudden cardiac death. Given the prognostic impact of these conditions, it is important to evaluate not only mitral valve morphology and regurgitation, but also the presence of left ventricular (LV) function and remodeling. To date, several possible hypotheses have been proposed regarding the underlying mechanisms of LV remodeling in the context of non-syndromic MVP, but the exact pathophysiological explanation remains elusive. Overall, volume overload related to severe MR is considered the main cause of LV dilatation in MVP. However, significant LV remodeling has been observed in patients with MVP and no/mild MR, particularly in patients with bileaflet MVP or Barlow’s disease, generating several new hypotheses. Recently, the concept of “prolapse volume” was introduced, adding a significant volume load to the LV on top of the transvalvular MR volume. Another possible hypothesis is the existence of a concomitant cardiomyopathy, supported by the link between MVP and myocardial fibrosis. The origin of this cardiomyopathy could be either genetic, a second hit (e.g., on top of genetic predisposition) and/or frequent ventricular ectopic beats. This review provides an overview of the different mechanisms and remaining questions regarding LV remodeling in non-syndromic MVP. Since technical specifications of imaging modalities impact the evaluation of MR severity and LV remodeling, and therefore might influence clinical decision making in these patients, this review will also discuss assessment of MVP using different imaging modalities.

## Introduction

Mitral valve prolapse (MVP) is a common valvular disorder with a prevalence of 2–3% in the general population (1).

In general, two main MVP subtypes can be distinguished that represent two ends of a disease spectrum in MVP. At one end, Barlow’s disease (BD) occurs in relatively young patients (20–40 years) and is characterized by dilatation of the mitral annulus and the elongation, thickening and prolapse of both leaflets, often associated with mitral annular disjunction. At the other end, fibroelastic deficiency (FED) occurs in older patients (50–70 years) and is characterized by single leaflet or segment prolapse, chordal elongation or rupture, and thickening of the prolapsing leaflet segments (2–4). Although some patients remain asymptomatic for a long time, MVP can be associated with mitral regurgitation (MR), LV dysfunction and remodeling with heart failure, ventricular arrhythmias and sudden cardiac death (5, 6).

Left ventricular (LV) remodeling and dysfunction are important features of disease progression and worse prognosis in many cardiovascular diseases, including MVP. The underlying mechanisms of LV remodeling in MVP are only partly understood and the exact pathophysiological process remains elusive. In general, volume overload related to MR is considered the main mechanism of LV remodeling in MVP. Therefore, current guidelines recommend surgical mitral intervention in severe MR (7, 8).

However, this concept has been challenged by the observation that LV dilatation and dysfunction can be disproportionate to the degree of MR, especially in patients with BD (9–11). Disproportionate LV remodeling in MVP can be defined as LV dilatation over the age- and gender-specific upper limit of normal when corrected for MR volume. Recently, several hypotheses have been introduced to explain this disproportionate LV remodeling in MVP, including additional volume overload by the prolapse volume (12), concomitant cardiomyopathy associated with myocardial fibrosis and ventricular arrhythmias (13–15) or genetic predisposition (16). Besides syndromic forms of MVP (e.g., Marfan syndrome), there is a growing body of evidence that non-syndromic MVP is also a genetic disease, characterized by autosomal dominant (17–20) or X-linked inheritance (21).

This review will provide an overview of the proposed mechanisms regarding LV remodeling in non-syndromic MVP. Furthermore, the assessment of MVP using different imaging modalities will be discussed as their technical specifications impact the evaluation of MR severity and LV remodeling, and therefore contribute substantially to clinical decision making in these patients.

## Mechanisms of Global Left Ventricular Remodeling in Mitral Valve Prolapse

### Left Ventricular Remodeling Related to Volume Overload

#### Mitral Regurgitant Volume

**FIGURE 1:**
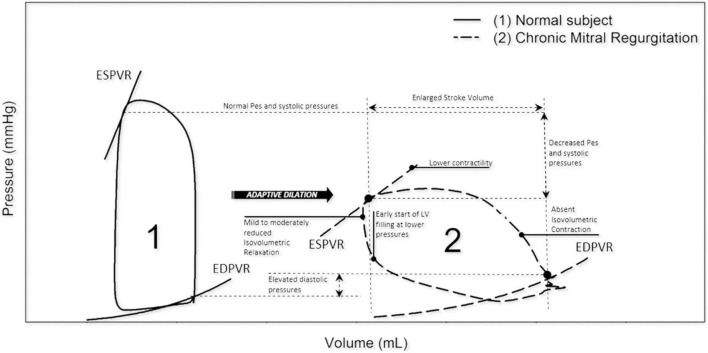
Left ventricle pressure-volume loops in normal vs. chronic MR. In chronic MR the LV pressure-volume loop shows a rightward shift toward larger ventricular volumes with increased total stroke volume. Note that isovolumetric contraction is absent and isovolumetric relaxation is reduced. EDPVR, end-diastolic pressure-volume relation; ESPVR, end-systolic pressure-volume relation; LV, left ventricular; MR, mitral regurgitation; Pes, end-systolic pressure. Reproduced from “Invasive left ventricle pressure–volume analysis: overview and practical clinical implications” by Bastos et al. (120) Copyright by © The Author(s) 2019. Distributed under the terms of Creative Commons Attribution Non-commercial License.

It has been established that severe primary MR (including due to MVP) can present with significant hemodynamic consequences, particularly on the LV and left atrium (LA) (Figure 1). More specifically, LA and LV remodeling or dilatation may occur in patients with chronic MR to compensate for the increased volume load and to maintain the forward stroke volume. In this chronic compensated phase, LV ejection fraction (LVEF) is (supra) normal and LA pressure is usually not elevated. Over time, this may progress to a chronic decompensated phase characterized by a decrease in forward stroke volume, rise in LA pressure and pulmonary hypertension. Despite development of significant myocardial dysfunction, LVEF may appear preserved due to low afterload in severe MR (22). In accordance with this theory, current guideline-based clinical practice focuses on volume overload related to chronic severe MR as the main mechanism of LV dilatation in patients with MVP (7, 8). LA and LV remodeling in primary MR should be differentiated from remodeling in secondary MR, as this form of MR typically occurs as a result of significant atrial (23) or ventricular (24) dilatation, e.g., in the presence of atrial fibrillation or dilated cardiomyopathy (25).

#### Prolapse Volume

While volume overload related to severe MR is considered the main cause of LV dilatation in MVP, several studies have challenged this concept by reporting significant LV dilatation in patients with MVP without a significant degree of MR (9–11). Yiginer et al. (10) reported LV enlargement in a small cohort of patients with classic bileaflet MVP in the absence of significant MR. Likewise, Malev et al. (9) detected increased LV dimensions and lower global longitudinal strain in patients with classic MVP without MR. Finally, Yang et al. (11) confirmed these findings in a larger cohort of patients with less than moderate MR with (*n* = 253) and without MVP (*n* = 344) and found that more severe LV remodeling was independently associated with MVP.

**FIGURE 2:**
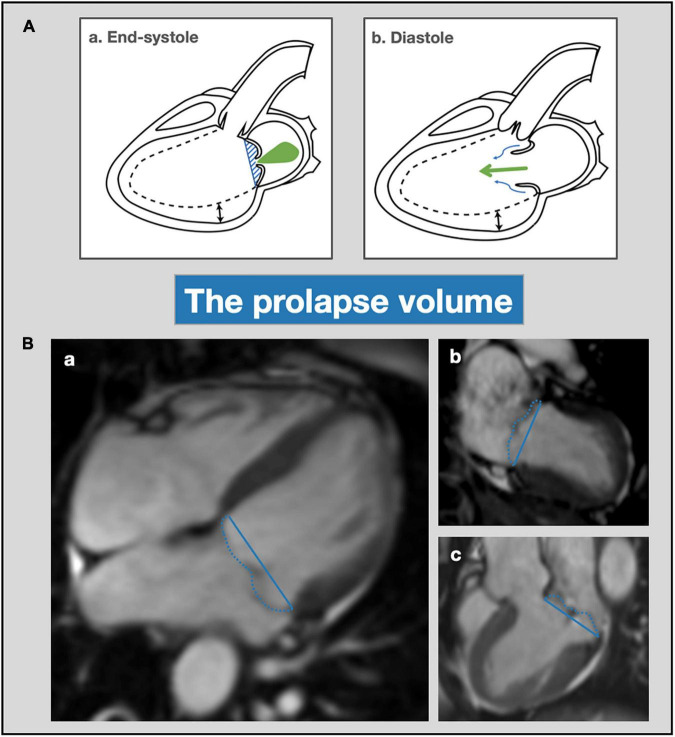
Prolapse volume—mechanism and assessment with cardiac magnetic resonance imaging. **(A)** Schematic overview of prolapse volume as a mechanism for progressive LV remodeling (dotted line). In end-systole (a) transvalvular MR occurs (green jet) and the prolapse volume is contained beneath the mitral leaflets (blue shaded area) and delineated by the mitral annulus (blue line). In diastole (b) the prolapse volume (blue arrows) exerts an additional volume load on the left ventricle on top of the transvalvular MR volume (green arrows). **(B)** Mitral valve prolapse volume assessment with CMR. CMR cine images of patient with bileaflet mitral valve prolapse and mitral regurgitation; 4-chamber (a), 2-chamber (b) and 3-chamber (c) views. Calculation of the prolapse volume is performed as previously described (12, 26). First, the mitral valve annulus diameter is measured in each view (indicated with the blue line) in order to calculate the average mitral annulus diameter. Then, the end-systolic prolapse area is measured by tracing the area between the mitral valve leaflets and annulus in each view (dashed blue line). By dividing the prolapse area by the annulus diameters, the prolapse height can be calculated for each view. Finally, the prolapse volume can be determined by multiplying mean prolapse height with annulus area, which can be calculated using the ellipse area formula. LV, left ventricular; MR, mitral regurgitation; CMR, cardiac magnetic resonance imaging.

A possible explanation for disproportionate LV remodeling relative to the degree of MR severity was recently proposed by El-Tallawi et al. (12). This study suggests that the total LV volume load in MVP is the sum of the transvalvular MR volume and the prolapse volume, which is defined as the end-systolic volume between the mitral annular plane and prolapsing leaflets (26) (Figure 2A). Especially in patients with BD and mitral annulus dilatation, the prolapse volume may contribute significantly to the total volume load. El-Tallawi et al. used cardiovascular magnetic resonance (CMR) to compare MR, prolapse volume and LV remodeling parameters in 157 patients with bileaflet prolapse (BD) or single leaflet prolapse. Despite similar transvalvular MR volumes, BD patients had significantly larger LV volumes. In addition, the prolapse volume (15.7 mL vs. 3.3 mL, *p* < 0.001) and therefore, the total volume load (59 mL vs. 42.5 mL, *p* < 0.001) were significantly larger in BD. A large prolapse volume of > 20 mL was present in 28% of BD patients. Furthermore, using the total volume load instead of transvalvular MR volume improved the correlation with LV end-diastolic volume in patients with BD, supporting the authors’ concept that disproportionate LV remodeling in BD can be explained by the total volume load (12). Recently, similar findings regarding the influence of prolapse volume on LV remodeling were reported by Levy et al. who also used CMR to compare myxomatous MVP (≈BD) with FED (27). In addition, Luyten et al. have confirmed the association of a large prolapse volume with significant LA and LV dilatation using echocardiography in patients with MVP and mitral annular disjunction (28).

#### Impact at the Cellular and Molecular Level

On a microscopic level, LV remodeling related to chronic LV volume overload is characterized by significant changes in the extracellular matrix, including alterations in collagen and matrix metalloproteinase expression (29–31). In addition, animal models have shown that extracellular matrix turnover and associated structural LV remodeling can be induced by mast cell activation and degranulation, e.g., by upregulation of the pro-inflammatory cytokine TNF-α (32–35). Furthermore, ventricular remodeling due to chronic volume overload has been associated with overexpression of transforming growth factor beta (TGF-β), which induces myocardial fibrosis through activation of fibroblasts and collagen synthesis (36). These findings need to be confirmed in human tissue to identify potential biomarkers for disease progression and it currently remains uncertain whether these molecular changes have a causative or compensatory role in the different stages of LV remodeling related to MR in MVP.

### Left Ventricular Remodeling Related to an Underlying Cardiomyopathy

Another theory to explain LV remodeling in MVP, certainly when disproportionate to the degree of MR, is the existence of an underlying myocardial disease—a cardiomyopathy. While the hypothesis of MVP-related cardiomyopathy has gained a lot of interest lately, this concept was first introduced several decades ago (37, 38). Early studies found an association of MVP with LV dysfunction (37), arrhythmias (39) and histologic evidence of myocardial fibrosis (38), suggestive of a concomitant myocardial disease. However, it is important to mention that quantification of MR and LV volumes was performed using ventriculography in these early studies, meaning that precise evaluation in accordance with current guidelines was not possible. In addition, these studies usually did not specify the MVP subtype as BD or FED.

**FIGURE 3:**
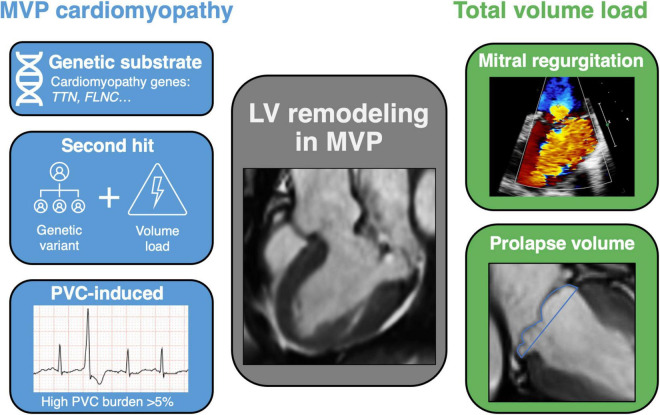
Central image: mechanisms of left ventricular remodeling in mitral valve prolapse. Schematic view of LV remodeling mechanisms in MVP, addressing the hypothesis of a cardiomyopathy on the left and total volume load on the right. Different pathophysiological mechanisms exist regarding the development of a cardiomyopathy in MVP, including a genetic substrate, second hit phenomenon and PVC-induced remodeling. On the other hand, the excessive volume load of chronic MR itself can induce LV dilatation, especially when a significant prolapse volume is added to the total volume load. LV, left ventricular; MVP, mitral valve prolapse; MR, mitral regurgitation; CMR, cardiac magnetic resonance imaging; *TTN*, titin; *FLNC*, filamin C; PVC, premature ventricular contraction.

At present, there are several possible hypotheses regarding the etiology of a concomitant cardiomyopathy in MVP (Figure 3).

#### Genetic Substrate

MVP is known to be associated with several connective tissue disorders such as Marfan syndrome and Loeys-Dietz syndrome, which are beyond the scope of this review (3, 40). Accordingly, it has been suggested that non-syndromic MVP could be correlated with connective tissue diseases as well.

Similar to syndromic forms of MVP, familial inheritance by autosomal dominant (17–20) and X-linked (21) transmission has been observed in non-syndromic MVP. To date, several (candidate) genes have been identified through linkage analysis and genome wide association studies, such as *DCHS1* (dachsous cadherin-related 1) (41), *TNS1* (tensin 1) (20), *LMCD1* (LIM and cysteine rich domains 1) (20), *DZIP1* (DAZ interacting zinc finger protein 1) (42), *GLIS1* (GLIS family zinc finger 1) (43) and *FLNA* (filamin A) (21). Interestingly, a very recent meta-analysis of six genome wide association studies identified several new candidate genes associated with MVP, including genes involved in TGF-β signaling and cardiomyopathy (44). While these genes have been related to the etiology of MVP, it is currently unclear whether they also act as a genetic substrate for disproportionate LV remodeling.

The recent association of MVP with pathogenic variants in several known cardiomyopathy genes, such as *TTN* (titin) and *FLNC* (filamin C) could explain disproportionate LV remodeling (16, 45). In 2020, Van Wijngaarden et al. (16) performed an extensive cardiac gene panel in 101 patients with MVP, predominantly BD phenotype (*n* = 96, 97%), in order to evaluate the genetic yield of the known causative MVP genes and detect possible new genetic variants. Interestingly, only 1 patient (1%) had a likely pathogenic variant in one of the causative MVP genes (*DCHS1*), but in 8 probands (8%) a likely pathogenic variant in four cardiomyopathy genes was observed (*DSP*, *HCN4*, *MYH6* and *TTN*), suggesting a common genetic foundation in the development of both myocardial and mitral valve disease. Most prevalent were *TTN* truncating variants (*n* = 5) which encode for the giant sarcomeric protein titin and are known to explain ca. 25% of patients with familial dilated cardiomyopathy (46). Furthermore, several case reports demonstrated a link between arrhythmogenic bileaflet MVP and truncating variants in the *FLNC* gene, which encodes an actin-binding protein associated with both hypertrophic and dilated cardiomyopathies (45). To date, the genetic substrate of MVP has not been linked with MR severity or LV remodeling parameters, so additional studies will be needed to elucidate the genotype-phenotype correlation and determine the role of familial screening in the future.

Furthermore, an MVP-related cardiomyopathy could be induced only in patients with a genetic substrate and an additional environmental risk factor that acts as a second hit. This mechanism has been described in dilated cardiomyopathy secondary to pregnancy, alcohol and anthracyclines (47). Most commonly, the culprit mutations that serve as a first hit are related to known cardiomyopathy genes, again the *TTN* gene in particular (48–50). Therefore, in the presence of a genetic substrate, the MR volume in MVP could act as a second hit to cause a dilated cardiomyopathy. In addition, prolapsing leaflets generate increased mechanical stress on the LV myocardium and papillary muscles which could induce focal fibrosis (51).

#### Arrhythmogenic

Supraventricular and ventricular arrhythmias have been established as a possible trigger for the development of a non-ischemic cardiomyopathy, characterized by reversible LV dysfunction and dilatation (52). In addition to tachycardia-induced cardiomyopathy, for example due to atrial fibrillation, frequent premature ventricular contractions (PVCs) are now recognized as a separate etiology of dilated cardiomyopathy (53). Whereas PVCs are frequently referred to as benign, even a low PVC-burden has been associated with increased risk of heart failure and LV dysfunction (54). The exact pathophysiology of PVC-induced cardiomyopathy is still unclear, but potential mechanisms are LV dyssynchrony and post-extrasystolic potentiation with Ca^2+^ overload (55–57).

Several studies have already demonstrated a correlation between MVP, ventricular arrhythmias and sudden cardiac death (15, 58). Complex PVCs, frequently arising from the papillary muscles or outflow tract, may induce LV dysfunction and act as a trigger for ventricular fibrillation as well (59). A significant improvement in LVEF has been observed after suppression of PVC burden with successful catheter ablation in patients with MVP, supporting the hypothesis of a PVC-induced cardiomyopathy (59, 60). Furthermore, Essayagh et al. showed that ventricular arrhythmias, defined as PVC burden >5%, ventricular tachycardia and ventricular fibrillation, are not only associated with LV dysfunction but with LA and LV dilatation as well (58). Therefore, the presence of PVCs or more complex arrhythmias appears to be an important risk factor in the process of disproportionate LV remodeling in MVP and screening with Holter monitoring should be considered.

## Mechanisms of Regional Left Ventricular Remodeling in Mitral Valve Prolapse

Apart from global LV remodeling, several regional LV remodeling patterns have been described in MVP.

First, focal LV hypertrophy has been observed in the basal inferolateral myocardial wall of patients with MVP, frequently coinciding with replacement fibrosis (15, 61), and the extent of basal hypertrophy has been correlated with the degree of mitral valve leaflet excursion (62). This regional hypertrophy may be triggered by dilatation of the LV base, especially in patients with mitral annular dilatation, due to increased wall tension by Laplace’s law (63). Furthermore, early angiographic studies have described a reduction in LV basal wall contractility, the so-called ballerina-foot pattern (37). These regional wall motion abnormalities have been confirmed more recently using speckle-tracking echocardiography and correlated with LV dilatation, mitral annular dilation and mitral annular disjunction (63–65). In addition to excessive regional stretch induced by posterior leaflet prolapse, abnormal contractility of the LV basal wall is thought to cause the typical systolic curling motion of the mitral annulus that is frequently referred to in patients with MVP (66, 67).

Second, it was recently hypothesized that morphological variations in mitral valve apparatus components, such as insertion of the papillary muscles, could alter (regional) LV remodeling. Moura-Ferreira et al. (68) found that apical insertion of the papillary muscles in patients with MVP induces significant changes in regional LV remodeling, such as focal thinning of the mid lateral wall and a lower global circumferential strain at this level, however, no changes in ventricular volumes or LVEF were observed. Furthermore, the prevalence of papillary muscle fibrosis was significantly higher in patients with apical papillary muscle insertion, presumably due to increased systolic traction and higher contractile force on the myocardium in these patients. Interestingly, they observed a higher burden of PVC’s and non-sustained ventricular tachycardias in patients with apical insertion of the papillary muscles (68).

Finally, several other LV abnormalities have been described in patients with MVP, such as LV non-compaction (69) and asymmetric septal hypertrophy (70), however these were only reported in small series.

## Mitral Valve Prolapse Subtypes and their Impact on Left Ventricular Remodeling

In MVP, two phenotypes can generally be distinguished—Barlow’s Disease (BD) and fibroelastic deficiency (FED). While current guidelines still recommend the same assessment and treatment for both entities (7, 8), they present with important differences in histopathology, echocardiographic characteristics and arrhythmogenic risk (2).

The pathophysiology of MVP is based on myxomatous degeneration of the valve, characterized by progressive thickening and increased area of the mitral valve leaflets. The normal valve tissue consists of 3 layers: the atrialis on the atrial side, the spongiosa as a middle layer and the fibrosa on the ventricular side (5). Histopathological analysis of the mitral valve identified that BD valves are characterized by expansion of the spongiosa layer due to proteoglycan accumulation and intimal thickening of fibrosa and atrialis (71, 72). This process of myxomatous infiltration causes leaflet thickening in BD. In contrast, FED showed more leaflet thinning due to impaired production of connective tissue with deficiency of collagen, elastin and proteoglycans. However, local thickening of the prolapsing segment can be observed in FED as well (71).

Presentation of BD is frequently in asymptomatic, younger patients (< 40 years) whereas FED occurs at a more advanced age (50–70 years), e.g., after chordal rupture (4). Interestingly, Hiemstra et al. observed that patients with BD more frequently report a familial history of primary MR compared to FED (26 vs. 8%) (73). Complex ventricular arrhythmias, ranging from ventricular ectopy to sustained ventricular tachycardia or even sudden cardiac death, have been associated particularly with bileaflet prolapse (≈BD) (13, 58).

Furthermore, it seems that bileaflet myxomatous MVP or BD is the main phenotype in patients with LV remodeling disproportionate to the degree of MR. In 2012, Yiginer et al. observed LV enlargement in classic bileaflet MVP even in the absence of significant MR (10). Similar findings were reported by Malev et al. (9), who studied 78 young adults with MVP without MR, and detected lower global longitudinal strain and larger LV dimensions in patients with classic prolapse compared to non-classic prolapse. In contrast, Yang et al. found early LV remodeling in patients with MVP and less than moderate MR severity, but observed no significant difference in chamber remodeling parameters between single and bileaflet prolapse (11). Overall, the role of MVP subtype remains debatable.

## The Elusive Link Between Left Ventricular Remodeling and Myocardial Fibrosis in Mitral Valve Prolapse

An important prognostic factor in the process of LV remodeling is the presence of myocardial fibrosis, which might be focal (replacement fibrosis) or diffuse (interstitial fibrosis). In patients with MVP, focal myocardial fibrosis has been observed mainly in the basal inferolateral LV wall and papillary muscles through histopathology (15, 51, 74) or by using CMR with late gadolinium enhancement (LGE) (14, 15, 51) and an association with malignant arrhythmias and sudden cardiac death has been established (13–15, 74). Interestingly, a CMR-based analysis of patients with chronic primary MR detected a significantly higher prevalence of focal LV fibrosis in MVP compared to non-MVP MR patients (36.7 vs. 6.7%; *p* < 0.001), suggesting a unique pathophysiological mechanism beyond MR causing LV fibrosis in MVP (61). Furthermore, the presence of LGE has been associated with LV dilatation in different cohorts of primary MR patients (61, 75). These findings have been validated in a population of patients with MVP by Constant Dit Beaufils et al. (14). Moreover, they observed focal LV fibrosis even in the absence of significant MR. In addition, subgroup analysis of patients with trace-mild MR (*n* = 120) showed LV dilatation (16%) and ventricular arrhythmias (25%), even in the absence of significant volume overload, suggesting another pathophysiological mechanism (14).

Besides focal fibrosis, limited data are available regarding the presence of diffuse interstitial fibrosis in patients with MVP, which can be associated with diastolic and systolic impairment. Two studies have shown that interstitial myocardial fibrosis as quantified with T1-mapping by CMR is correlated with LV dilatation in primary MR (76), and more specifically in MVP (77). Although it is assumed that chronic MR with LV volume overload leads to diffuse myocardial fibrosis which may ultimately result in heart failure, some evidence points toward a more specific myocardial disease in MVP. Bui et al. showed that interstitial fibrosis as assessed by T1-mapping was not related to MR severity in patients with MVP, however, this study may be underpowered due to the small sample size (77). In contrast, Kitkungvan et al. recently demonstrated that the presence of diffuse interstitial fibrosis was associated with increase in MR severity, but not with MVP in particular (78). In the future, large multicenter studies will be required to further evaluate the role of T1-mapping in the risk stratification of patients with primary MR and MVP.

## Left Ventricular Reverse Remodeling

Several studies have demonstrated that surgical correction of severe primary MR can reverse the process of LV remodeling by eliminating chronic volume overload (79–81). The phased process of LV reverse remodeling, characterized by a decrease in LV dimensions and improvement of LV systolic function, was recently investigated by Le Tourneau et al. (81) using echocardiographic follow-up after mitral valve surgery. The initial response after surgery is a significant decrease in LV end-diastolic volume and LVEF which largely depends on the pre-operative regurgitant volume. After several months, a decrease in LV end-systolic volume and improvement of LVEF can be observed (81). Besides pre-operative MR severity, other determinants of LV reverse remodeling and normalization of LVEF are preserved LVEF and smaller LV dimensions at baseline (82, 83). Interestingly, a recent CMR study in patients with primary MR showed that mitral valve surgery can even result in reverse myocardial remodeling with a reduction in diffuse myocardial fibrosis (84).

In MVP specifically, a similar evolution with decrease in LV volumes has been demonstrated following mitral valve repair or replacement (82, 85, 86). As mentioned earlier, the total LV volume load in MVP consists of the transvalvular MR volume and the prolapse volume, which can be significant especially in bileaflet prolapse. We could therefore hypothesize that LV reverse remodeling in MVP will be optimal if the prolapse volume is corrected as well, e.g., by mitral annuloplasty and partial leaflet resection if needed in contrast to an Alfieri procedure without annuloplasty, although there are currently no data on this specific topic. Of note, a recent study by Essayagh et al. (87) showed that LV remodeling post-mitral valve repair in patients with MVP was similar between patients with and without mitral annular disjunction—which is related to the height of the prolapse volume—if the disjunction was corrected.

## Assessment of Mitral Valve Prolapse, Mitral Regurgitation Severity and Left Ventricular Remodeling by Cardiovascular Imaging

Any study investigating MVP and LV remodeling is impacted by the limitations of the imaging method used. Therefore, clinicians should be aware of particular limitations and strengths of each modality when assessing a patient with MVP, summarized in Table 1.

**TABLE 1:** Strengths and limitations of echocardiography vs. cardiac magnetic resonance imaging (CMR) in the assessment of mitral valve prolapse.

	Echocardiography		CMR	
	+	–	+	–
General	Widely available Evaluation of prolapsing leaflet/segment and MVP subtype Hemodynamic impact (filling pressures and pulmonary hypertension)	TTE: limited by poor echogenicity TEE: semi-invasive technique	Tissue characterization (T1 mapping, LGE) Accurate quantification of prolapse volume (26)	Relative contra-indications: incompatible implanted devices, claustrophobia,… Inaccurate measurements with arrhythmia or poor breath-holding
MR quantification	Greater sensitivity (small jets)	Mid-late systolic MR: overestimation of severity with PISA and EROA (79, 89) Eccentric jet difficult to quantify High intra- and interobserver variability (114, 115)	Lower intra- and interobserver variability (116, 117) Better correlation with outcome (110, 118)	No well-established cut-off to define severe MR Indirect method: includes prolapse volume in MR volume (100)
LV remodeling	Quick evaluation of LV volumes and function	Underestimation of LV volumes and overestimation of LV ejection fraction compared to CMR (2D > 3D echo) (119)	More accurate assessment of LV volumes (gold standard) (96, 97)	LV volumes and ejection fraction dependent on definition of LV base (100)

### Echocardiography

#### Transthoracic Echocardiography

Routine 2D TTE is generally the first-line imaging tool to diagnose MVP and usually allows for a correct identification of the prolapsing leaflet segments. However, it is important to note that the diagnosis of MVP should be made in the parasternal (or apical) long-axis view but not in the apical four-chamber view because the saddle-shaped annulus could lead to false positive diagnosis (88).

**FIGURE 4:**
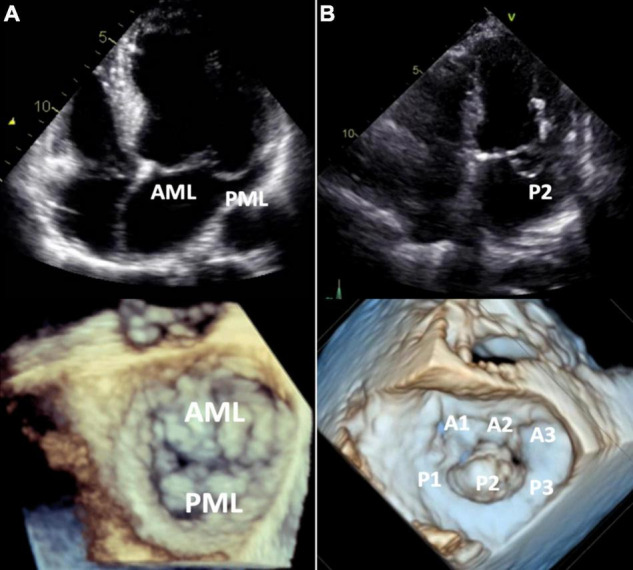
Barlow’s disease vs. fibroelastic deficiency. 2D transthoracic four-chamber view (upper panels) and 3D transesophageal focused view of the mitral valve (lower panels). **(A)** Barlow’s disease with annular dilatation, thickened leaflets and bileaflet prolapse (anterior + posterior mitral leaflet). **(B)** Fibro-elastic deficiency with prolapse (flail) of the P2 segment of the posterior mitral leaflet due to chordal rupture. Mitral annulus diameter is normal in this case, but mild annular dilatation can be present. AML, anterior mitral leaflet; PML, posterior mitral leaflet.

Following diagnosis of prolapse, MVP subtypes (BD vs. FED) can be differentiated by comprehensive echocardiographic assessment (Figure 4).

**FIGURE 5:**
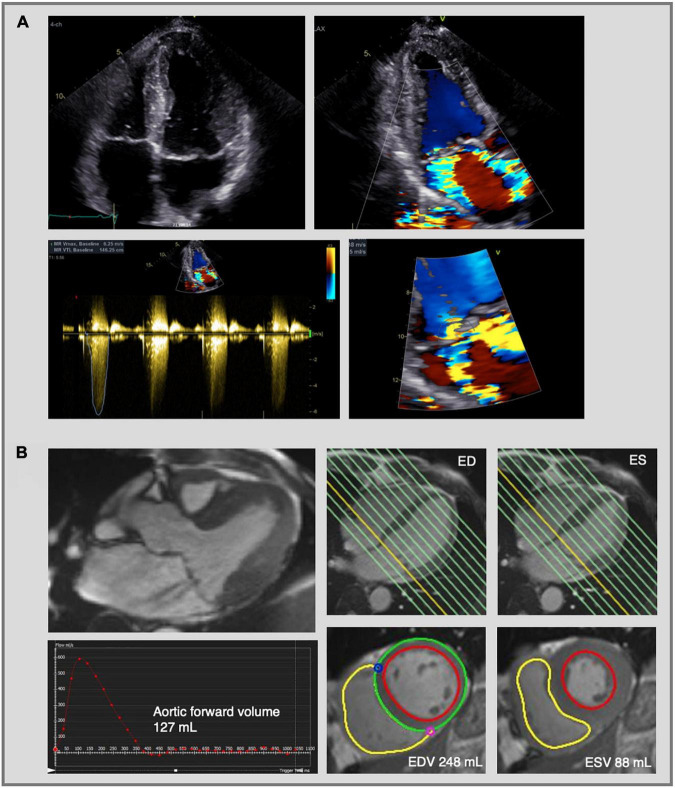
Mitral regurgitation assessment by echocardiography and cardiac magnetic resonance imaging. Complex evaluation of moderate-severe mitral regurgitation (MR) with multiple jets in a patients with bileaflet mitral valve prolapse. **(A)** 2D transthoracic echocardiography using color flow Doppler shows two MR jets—an eccentric jet toward the intra-atrial septum and a jet toward the lateral wall. The PISA radius was measured at 8 mm, but given the multiple and eccentric jets, this parameter is not reliable to calculate the EROA and MR volume, which indicates the need for further evaluation using CMR. **(B)** CMR images of the same patient as in panel **(A)**. Three-chamber cine image shows the presence of MR with two jets (upper left image). MR volume was calculated at 33 mL using the indirect method based on the difference between LV stroke volume (EDV—ESV = 160 mL) and aortic forward volume (127 mL). The LV stroke volume was calculated from the short-axis cine images in end-diastole and end-systole (lower right images) and the aortic flow was calculated by phase-contrast imaging (lower left image). Traditionally the LV base is defined at the level of the mitral valve annulus (yellow line, upper right images) and therefore the calculated regurgitant volume includes both the transvalvular MR volume and the prolapse volume. MR, mitral regurgitation; PISA, proximal isovelocity surface area; EROA, effective regurgitant orifice area; CMR, cardiac magnetic resonance imaging; LV, left ventricle; ED, end-diastolic; ES, end-systolic; EDV, end-diastolic volume; ESV, end-systolic volume.

As per international society recommendations, echocardiographic assessment of MR severity should be performed using a multi-integrative approach including both qualitative and quantitative parameters (7, 8) (Figure 5A). In the clinical evaluation of patients with valve regurgitation, it is critical to differentiate severe from non-severe MR since the former may implicate the need for (surgical) intervention. When feasible, the proximal isovelocity surface area (PISA) method is recommended to quantify the regurgitant volume and effective regurgitant orifice area (EROA) (22).

While 2D TTE is widely used as the principal technique to investigate MR severity, several limitations have to be addressed, which can originate from specific MR characteristics, such as orifice morphology, eccentric or multiple jets… (Table 1). For example, patients with mid-late systolic MR might have a similar jet area and EROA compared to holosystolic MR, but a lower regurgitant volume (and therefore more benign outcome) due to the shorter regurgitant time interval (89). Correspondingly, the PISA radius may be variable during the cardiac cycle and increase during systole to reach a maximum in mid- to end-systole. It is important to note that not considering these limitative factors of Transthoracic Echocardiography (TTE) could lead to an overestimation of MR severity and possibly even excess surgical interventions.

Echocardiographic evaluation of LA volume and LV dimensions, LVEF and systolic pulmonary pressure is recommended in all patients with more than mild MR (22). In the context of MR, LV dilatation is defined as LV end-systolic dimension ≥ 40 mm (7, 8), which is less load-dependent compared to LV end-diastolic dimension. After correction for body surface area, LV end-systolic volume and end-diastolic volume can provide more insight in the degree of ventricular remodeling. In order to detect early LV dysfunction, global longitudinal strain can be considered (90, 91). Importantly, 3D echocardiography has superior accuracy compared to 2D echocardiography regarding the evaluation of LV volumes and LVEF, as it avoids foreshortening of the LV (92). The prolapse volume can be quantified using 2D TTE (28) and 3D TEE (93), although this measurement is currently not part of standard clinical practice.

#### Transesophageal Echocardiography

Part of the limitations of TTE, such as poor echogenicity, can be overcome by using Transesophageal Echocardiography (TEE). In addition, TEE can provide a more detailed evaluation of valve geometry and dynamics, and help to characterize BD vs. FED. Benefits like higher resolution, multiplane and proximity to the mitral valve enhance MR evaluation techniques such as PISA and vena contracta compared to TTE (94). Moreover, 3D echocardiography allows for direct delineation of the vena contracta and EROA, which may improve MR severity assessment, especially if the mitral regurgitant orifice is non-circular. Furthermore, the surgical view of the mitral valve can be visualized by 3D TEE and has a high specificity and sensitivity for the diagnosis of MVP. Consequently, current international guidelines state that 3D echocardiography should be incorporated in the clinical assessment of patients with particularly complex mitral valve pathology (92).

### Cardiac Magnetic Resonance Imaging

Due to the known limitations of echocardiography and therapeutic consequences of severe MR, CMR has emerged as an interesting non-invasive imaging modality for the evaluation of MVP morphology and MR severity (95). At present, CMR is generally indicated in patients with MVP when echocardiographic images are suboptimal, when there is discordance between MR severity by echocardiography and clinical findings, and to evaluate the presence of myocardial fibrosis (94). Importantly, CMR is currently considered as the gold standard for the assessment of atrial and ventricular volumes and function (96, 97).

Quantification of MR volume using CMR can be performed using direct or indirect methods. The most commonly used indirect CMR method relies on two techniques for the quantification of MR: phase-contrast imaging to measure aortic flow and short-axis cine images to quantify LV stroke volume (98, 99) (Figure 5B). In patients with lone MR, the LV stroke volume contains the forward stroke volume and mitral regurgitant volume. Thus, the MR volume can be calculated by subtracting the aortic forward flow from the total LV stroke volume. Importantly, in patients with MVP the LV base (at end-systole) is usually defined at the mitral valve annulus and not at the mitral valve leaflets, meaning that the calculated regurgitant volume includes the prolapse volume (100) (Figure 5B).

Moreover, CMR also allows direct quantification of MR by measuring the regurgitant flow over the mitral valve with phase-contrast imaging (101). Recently, four-dimensional (4D) flow CMR has emerged as an innovative imaging technique to quantitate MR based on a three-dimensional and time-resolved assessment of blood flow MR (102). Both in patients with primary MR and specifically in patients with MVP, quantification of mitral regurgitant volume has shown to be reproducible and feasible using 4D flow CMR (103, 104). However, this technique requires additional acquisition and post-processing time and future studies are needed to evaluate a potential clinical outcome benefit over the indirect 2D CMR technique (102).

In addition to transvalvular MR, the prolapse volume can represent a significant volume load and should be assessed in patients with MVP (12). The indirect CMR method to assess MR already incorporates the prolapse volume, therefore possibly overestimating MR severity in patients with severe bileaflet prolapse (26), while echocardiography and direct CMR methods only assess the transvalvular MR volume (Figure 5). Using CMR, the prolapse volume can be calculated from 4-, 3-, and 2-chamber views by measuring end-systolic mitral annulus diameter and prolapse area, as is shown in Figure 2B (26).

**FIGURE 6:**
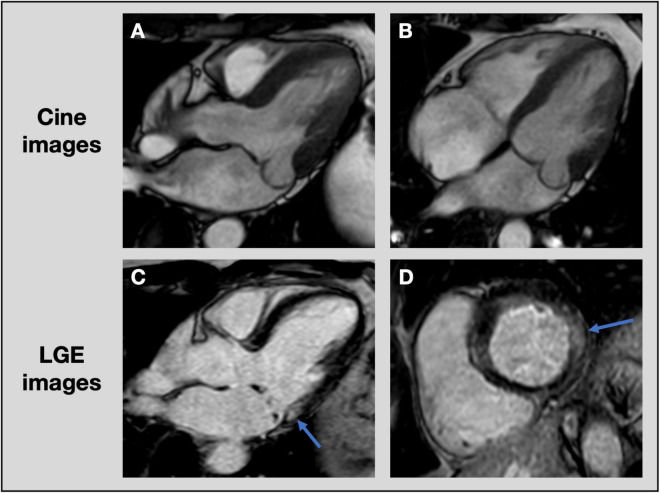
Assessment of focal fibrosis with cardiac magnetic resonance imaging. CMR cine images of patient with posterior leaflet prolapse on 3-chamber **(A)** and 4-chamber **(B)** views. Late gadolinium enhancement images show evidence of focal fibrosis in the basal inferolateral wall (blue arrows) on 3-chamber **(C)** and short axis **(D)** views. CMR, cardiac magnetic resonance imaging; LGE, late gadolinium enhancement.

An important additional benefit of CMR in MVP, beyond accurate quantification of MR and LV volumes, is the ability to detect focal or diffuse LV fibrosis. LGE CMR is the most accurate non-invasive technique to assess focal myocardial replacement fibrosis (Figure 6). Given the association of BD with ventricular arrhythmias and fibrosis, detection of LGE using CMR may improve risk stratification in these patients (13, 15, 61). In addition, T1 mapping can quantify diffuse interstitial fibrosis, demonstrated by an elevated native T1 time and extracellular volume expansion (105, 106). Although these CMR techniques are increasingly used in valvular heart disease and the extent of fibrosis is strongly associated with patient outcomes, there are currently no well-established cut-off values to refer patients for valve surgery (107).

## Future Directions

Although prognosis can be benign in many patients with MVP, poor outcomes have been observed in relation to severe MR, heart failure and malignant ventricular arrhythmias (13, 58). In large cohorts of patients with primary MR, severe LV remodeling with an increase in LV dimensions and decrease in LVEF was correlated with worse prognosis, but no comparison was made between MVP and non-MVP (108–110). Moreover, outcome studies that compare patients with MVP with and without disproportionate LV remodeling are still lacking. In order to optimize treatment options and improve patient outcomes, further studies are needed to provide more insight into these different mechanisms of LV remodeling, such as severe volume overload or a genetic cardiomyopathy.

First, to assess LV remodeling related to volume overload, a careful quantification of LV volumes and MR severity is crucial, emphasizing the importance of accurate cardiac imaging techniques. An important question that still remains is the clinical and prognostic importance of the total volume load, including both MR volume and prolapse volume, especially in patients with BD. Although mitral valve repair or replacement would reduce both MR volume and prolapse volume, there are currently insufficient data to base surgical indications on total volume load instead of MR severity alone. Furthermore, optimal risk assessment and timing of mitral valve surgery is still debated in patients with MVP. Postoperative LV reverse remodeling has been observed to be less favorable in patients with LV dilatation and dysfunction prior to mitral valve surgery (81, 111). Therefore, some authors advocate for early intervention, in contrast with the current guidelines (112).

Second, if further studies confirm the hypothesis of a genetic cardiomyopathy in patients with MVP, it will be important to elucidate the genotype-phenotype relationship in order to refer patients for genetic counseling. In addition, this may provide the opportunity for familial screening, preclinical diagnosis and better follow-up.

Third, despite the established association of LGE on CMR with worse event-free survival in patients with MVP (14), the exact role of focal or diffuse LV fibrosis in the risk stratification for heart failure or malignant arrhythmias needs to be further explored. Since non-invasive detection of fibrosis is only possible using CMR, will this investigation be indicated for all patients with MVP in the future or primarily for those with the BD subtype or history of arrhythmias?

Finally, the presence of PVC’s and arrythmias are a known risk factor for sudden cardiac death and LV remodeling in patients with MVP. Therefore, screening with Holter monitoring should be considered. The possible benefit from radiofrequency ablation in patients with MVP to reverse a PVC-induced cardiomyopathy and decrease the risk of malignant arrhythmias needs to be investigated further (59, 60, 113).

## Conclusion

To conclude, severe LV dilatation and dysfunction are important markers of disease progression and may indicate worse prognosis and the need for mitral valve surgery in patients with MVP. An in-depth assessment of MR severity, LV volumes and function, and myocardial fibrosis by cardiac imaging techniques is crucial to determine patients at risk. In addition, insight in the potential mechanisms behind this process of LV remodeling is of great importance. While some interesting hypotheses have been proposed, it is currently still debated whether LV remodeling occurs only due to severe volume overload or whether an underlying cardiomyopathy may be the cause. At present, it is clear that many questions still remain unanswered and therefore large multicenter studies are needed to further elucidate mechanisms of LV remodeling, identify patients at risk and improve treatment and outcome in MVP.

## Author Contributions

LP, PB, and CV performed the data search and drafted the manuscript. BP, HH, and EV critically revised the draft. All authors contributed to the conceived of this work and approved the final version of the manuscript.

## Conflict of Interest

The authors declare that the research was conducted in the absence of any commercial or financial relationships that could be construed as a potential conflict of interest.

## Publisher’s Note

All claims expressed in this article are solely those of the authors and do not necessarily represent those of their affiliated organizations, or those of the publisher, the editors and the reviewers. Any product that may be evaluated in this article, or claim that may be made by its manufacturer, is not guaranteed or endorsed by the publisher.

## Abbreviations

BD: Barlow’s Disease
CMR: cardiac magnetic resonance
EF: ejection fraction
EROA: effective regurgitant orifice area
FED: fibroelastic deficiency
FLNC: filamin C
LA: left atrium
LGE: late gadolinium enhancement
LV: left ventricle
MR: mitral regurgitation
MVP: mitral valve prolapse
PISA: proximal isovelocity surface area
PVC: premature ventricular contraction
TGF- β: transforming growth factor beta
TTE: transthoracic echocardiography
TTN: titin
TEE: transesophageal echocardiography.
